# Interfacial Properties of Gold and Cobalt Oxyhydroxide in Plasmon-Mediated Oxygen Evolution Reaction

**DOI:** 10.1021/acs.jpcc.4c06632

**Published:** 2025-01-06

**Authors:** Janet Zhen, Timothy Lin, Tucker Forbes, Mark Engelhard, Jingjing Qiu

**Affiliations:** † Department of Chemistry and Biochemistry, San Francisco State University, 1600 Holloway Ave., San Francisco, California 94132, United States; ‡ Energy & Environment, Pacific Northwest National Laboratory, Richland, Washington 99354, United States

## Abstract

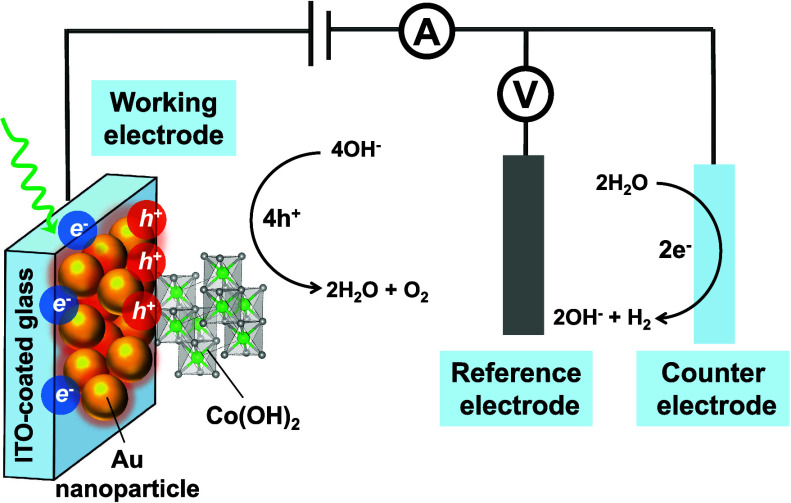

Water electrolysis is a green method of storing electrical energy in the chemical bonds of high-energy hydrogen gas (H_2_). However, the anodic oxygen evolution reaction (OER) requires a significant kinetic overpotential, limiting the electrolysis rate. Recently, plasmonic gold nanoparticles (Au NPs) have been introduced to improve charge transfer at the interface between the OER electrocatalysts and the electrolyte under light illumination. Despite this, the mechanism by which Au NPs enhance photoassisted electrochemical processes remains poorly understood. To address this, we employed a model system comprising a plasmonic Au electrode and a cobalt (Co)-based electrocatalyst in alkaline electrolytes, studying the plasmon-mediated OER process through (photo)electrochemical and spectroscopic methods. Our findings revealed that a surfactant-free, electrodeposited plasmonic Au electrode could significantly enhance the electrocatalytic performance of Co-based OER electrocatalysts under continuous visible and near-infrared light illumination. Transient photocurrent studies showed that both the photothermal effect and energetic charge carriers contributed to the improved OER performance, with the Au|Co catalyst interface playing a key role in these enhancements. Additionally, electrochemical Raman measurements identified the active phase of the Co-based OER electrocatalyst to be cobalt oxyhydroxide (CoOOH) at oxidizing potentials.

## Introduction

Water electrolysis offers a sustainable method for generating high-energy hydrogen (H_2_) fuel without producing carbon-based byproducts.^1^ H_2_ gas is produced on the cathode while oxygen (O_2_) gas evolves from the anode in this process. However, the overall water-splitting process is slowed due to the high activation energy of the oxygen evolution reaction (OER) compared to that of the hydrogen evolution reaction (HER) counterpart.

To improve the OER kinetics and lower the overpotential (η), a variety of electrocatalysts have been explored to boost the interfacial charge transfer and overall OER efficiencies.^2^ Among the most effective OER electrocatalysts is iridium oxide (IrO_2_), known for its high activity and relative stability in acidic electrolytes, though it is costly and scarce.^3^ In contrast, more affordable options include first-row transition metal-based oxides, hydroxides, and oxyhydroxides—such as those of nickel (Ni), cobalt (Co), iron (Fe), and manganese (Mn)—which are particularly active in alkaline electrolytes.^4−6^ To further optimize the efficiencies of these OER electrocatalysts, different strategies and fundamental studies have been pursued to elucidate the relationship between structure and performance, as well as key factors driving interfacial charge transfer.

Recently, it has emerged as an advanced strategy by incorporating plasmonic properties into electrocatalytic systems to achieve higher performance and/or selectivity of the reactions.^7^ Common plasmonic metals used in the electrocatalytic systems include gold (Au) and silver (Ag), as they can be excited with visible light,^8,9^ are conductive electrode materials, and sometimes act as efficient electrocatalysts. Plasmonic Au NPs have been explored in different electrocatalytic systems.^7,10−13^ For example, Au nanostructures such as spherical Au NPs, Au nanofiber, and porous Au electrode have been shown effective in mediating oxidation reactions of alcohol, water, and glucose.^10,11,14^ In addition to enhancing the reactions directly, Au NPs have also been shown to enhance the electrocatalytic performances of the OER reaction through an electrocatalyst.^13,15^ Although plasmonic properties have been shown to be useful in mediating electrocatalytic processes, the fundamental mechanism behind the enhancement is still under debate.^16^ The key point of debate is whether the photothermal effect or energetic (“hot”) charge carriers play the dominant role in the plasmon-enhanced processes.^16−20^ For the plasmon-mediated OER process, a key question is regarding how plasmonic nanostructures influence the reaction through the electrocatalyst, as the energy dissipation pathways can differ compared with when plasmonic nanostructures are used directly as the electrodes.

Surface plasmon resonance (SPR) is the collective oscillating electromagnetic radiation from free electrons when the plasmonic metals absorb resonant light.^21^ The SPR creates a population of energetic electrons and holes with a short lifetime on the femtosecond scale. Following the SPR excitation, the plasmon can dissipate energy via radiative or nonradiative decays. Radiative decay can transfer energy via light scattering and near-field coupling, while nonradiative decay eventually results in localized heating. In radiative decay, the local electromagnetic field is significantly enhanced, a phenomenon often utilized in surface-enhanced Raman scattering (SERS) applications.^22^ In the nonradiative decay pathway, energetic (“hot”) charge carriers can be harnessed for reactions if a suitable interface is available; otherwise, the energy dissipates as heat.^21,23^ The architecture and operating conditions of the system containing a plasmonic component could greatly affect the energy-dissipating channel as shown in previous studies.^23^ For example, photoelectrochemistry enables the localization of “hot” holes on photoanodes and offers an approach to explore the hole-transfer dynamics.^24^

To achieve a mechanistic understanding of how plasmons and the plasmonic metal assist the electrocatalytic OER process through an electrocatalyst, we investigated the impacts of plasmonic Au nanostructures on the cobalt oxyhydroxide (CoOOH) catalyzed OER process in alkaline electrolyte, which focuses on the interfaces between the plasmonic Au component and the OER electrocatalyst. Cobalt hydroxide (Co(OH)_2_) was prepared as the starting electrocatalyst system, and we applied the electrochemical Raman spectroscopy (EC-Raman) to further understand the surface changes of the electrocatalyst layer under different applied potentials. CoOOH is found as the active phase of this Co-based electrocatalyst at the OER potentials. Based on the knowledge of the electrocatalyst system, three different interfaces of Au nanostructures and CoOOH were designed and synthesized to compare their performances in the light-mediated OER process as the interfaces determine the lifetime of the energetic charge carriers from the SPR. Specifically, electrodeposited Au nanoflowers and Au nanofilms were used as the conductive plasmonic electrodes, and it is expected the energetic electrons are separated from the holes through the assistance of the external circuit. The CoOOH electrocatalyst in direct contact with such electrodes will harness the energetic holes more effectively. A control architecture in which CoOOH electrocatalysts were interfaced with free-flowing surfactant-coated Au nanoshells was adopted for comparison. These free-flowing Au nanoshells will create an indirect interface with the electrocatalyst and are expected to be less effective in separating the energetic charge carriers. These structures were characterized with scanning electron microscopy (SEM), X-ray photoelectron spectroscopy (XPS) and optical measurements. Cyclic voltammetry (CV) measurements and Tafel analysis under both dark and illumination conditions were applied to assess the OER efficiency of different electrodes and to evaluate the impact of plasmon on the reaction. Furthermore, transient photocurrents, light-power-dependent photocurrents, and bulk heating control experiments were performed to investigate the contribution of photothermal and energetic charge carrier (i.e., photoelectrical) effects from the plasmonic Au components.

## Experimental Section

### Chemicals

Chemicals and reagents used in this study include the following: potassium hydroxide (KOH, Sigma-Aldrich), sulfuric acid (H_2_SO_4_, Sigma-Aldrich), sodium chloride (NaCl, Sigma-Aldrich), tetrachloroauric acid (HAuCl_4_, Sigma-Aldrich), cobalt nitrate (Co(NO_3_)_2_, Sigma-Aldrich), PEG-coated silica core Au nanoshell solution (SiO_2_@Au, 0.05 mg/mL, nanoComposix), and cobalt oxide powder (Co_3_O_4_, Sigma-Aldrich). All chemicals were used as received without further purification. All solutions were prepared with 18.2 MΩ·cm DI water.

### SEM Measurements

The SEM images of the electrodes were acquired on a Carl Zeiss Ultra 55 Field Emission Scanning Electron Microscope (FE-SEM). The voltage applied for imaging was 1.50–2.00 keV.

### Optical Property Measurements

The optical responses of the electrodes were measured on a Cary 6000i UV–vis-NIR spectrometer (Agilent) in transmission mode. The substrates were mounted on a mask with a circular aperture with a diameter of 5 mm in a solid sample holder for fixed position transmittance measurements. The aperture allowed beam collimation and transmittance measurement of the samples.

### XPS

XPS measurements were performed with a Physical Electronics Quantera SXM Scanning X-ray Microprobe and a Thermo Fisher NEXSA spectrometer with a 125 mm mean radius, full 180° hemispherical analyzer, and 128-channel detector. This Physical Electronics system uses a focused monochromatic Al Kα X-ray (1486.7 eV) source for excitation and a spherical section analyzer. The instrument had a 32-element multichannel detection system. The X-ray beam is incident normal to the sample, and the photoelectron detector is at 45° off normal. High-energy resolution spectra were collected using a pass-energy of 69.0 eV with a step size of 0.125 eV. For the Ag 3d_5/2_ line, these conditions produced a fwhm of 0.92 eV ± 0.05 eV. The binding energy (BE) scale is calibrated using the Cu 2p_3/2_ feature at 932.62 ± 0.05 eV and Au 4f_7/2_ at 83.96 ± 0.05 eV. Low-energy electrons at ∼1 eV, 20 μA and low- energy Ar^+^ ions were used to minimize sample charging during analysis. The Thermo Fisher NEXSA spectrometer system uses a focused monochromatic Al Kα X-ray (1486.7 eV) source for excitation and an electron emission angle of 60°. The narrow scan spectra were collected using a pass-energy of 50 eV with a step size of 0.1 eV. For the Ag 3d_5/2_ line, these conditions produced a fwhm of 0.84 eV ± 0.02 eV. The BE scale is calibrated using the Cu 2p_3/2_ feature at 932.62 ± 0.05 eV and 4f_7/2_ feature at 83.96 ± 0.05 eV.

### Raman Measurements

The Raman and EC-Raman measurements were conducted with a Horiba TRIOS Tip-enhanced Raman Spectroscopy Microscope. The excitation laser is 532 nm with an incident laser power of 6 or 0.6 mW. The EC-Raman measurements were conducted in a 5 mL three-electrode electrochemical cell equipped with a quartz window (DekResearch). The objective lenses used were 100× in the dry Raman measurements and 10× in the EC-Raman measurements.

### Electrode Preparations

The indium tin oxide (ITO)-coated glass electrodes (Delta Technologies) were used as the conductive substrate. Electrodeposition of Au nanoflowers on the ITO electrode was carried out in an aqueous solution containing 3 mM HAuCl_4_ and 0.5 M H_2_SO_4_ at −0.20 V versus a Ag/AgCl reference electrode for 2 min following the protocol in a previous report.^16^ The electrodeposition of Au nanofilms was conducted in an aqueous solution of 5 mM HAuCl_4_ with 0.1 M NaCl of 10 s intervals at −0.2 and −0.6 V versus a Hg/HgO reference electrode and a Pt counter electrode modified from a previously reported protocol.^25^ 500 μL PEG-coated Au nanoshell solution with a concentration of 0.05 mg/mL and 660 nm absorbance maximum was added to the 0.1 M KOH electrolyte in the control experiments. Co(OH)_2_ depositions were performed in a 0.1 M Co(NO_3_)_2_ solution with a constant current density of −0.1 mA/cm^2^ following a previous report.^26^

### (Photo)electrochemical Measurements

All (photo)electrochemical experiments were performed with a BioLogic SP-300 potentiostat. The photoelectrochemical measurements were conducted in a 5 mL three-electrode electrochemical cell equipped with a quartz window (DekResearch) for top illumination. The electrochemical measurements of Co(OH)_2_ electrodes were conducted in a three-electrode glass cell. The working electrode (WE) was an ITO-coated glass (Delta Technologies) that was then electrodeposited with Au and/or Co(OH)_2_ layers. The counter electrode (CE) was a coiled platinum wire and the reference electrode (RE) used in the CV measurements was a Hg/HgO electrode (filled with 1 M KOH) or a Ag/AgCl electrode (filled with sat. KCl). The electrolyte for all (photo)electrochemical measurements was 0.1 M KOH. The conversion between Hg/HgO and reversible hydrogen electrode (RHE) references is *E* (vs RHE) = *E* (vs Hg/HgO) + 0.896 V in 0.1 M KOH, and the conversion between Ag/AgCl and RHE is *E* (vs RHE) = *E* (vs Ag/AgCl) + 0.9646 V in 0.1 M KOH. The light source applied for top illumination is a solar simulator (Abet) with a 515 nm long-pass filter to produce broadband visible and near-infrared light. The light source applied for the illumination of the PEG-coated Au nanoshell control experiment without the Co(OH)_2_ is a 625 nm LED light (Thor Lab). The light intensity of the illumination was measured using a power meter equipped with a thermopile sensor (Newport).

## Results and Discussion

The electrocatalyst Co(OH)_2_ has been shown to be effective in the OER under alkaline conditions, and O_2_ is expected to be the sole product under alkaline conditions during the Co-catalyzed heterogeneous catalysis.^27^ It has been suggested that the electrochemical transition from Co(OH)_2_ to CoOOH is key to catalyze the OER process, with the redox behaviors of the Co metal center playing an important role.^1,28^ In this study, the electrodeposited Co(OH)_2_ film with well-defined redox properties was used as a model system of the OER electrocatalyst. These films were interfaced with plasmonic Au electrodes or free-flowing Au nanoparticles to investigate how the plasmon influences the OER process with different Au|Co(OH)_2_ interfaces.

**Figure 1:**
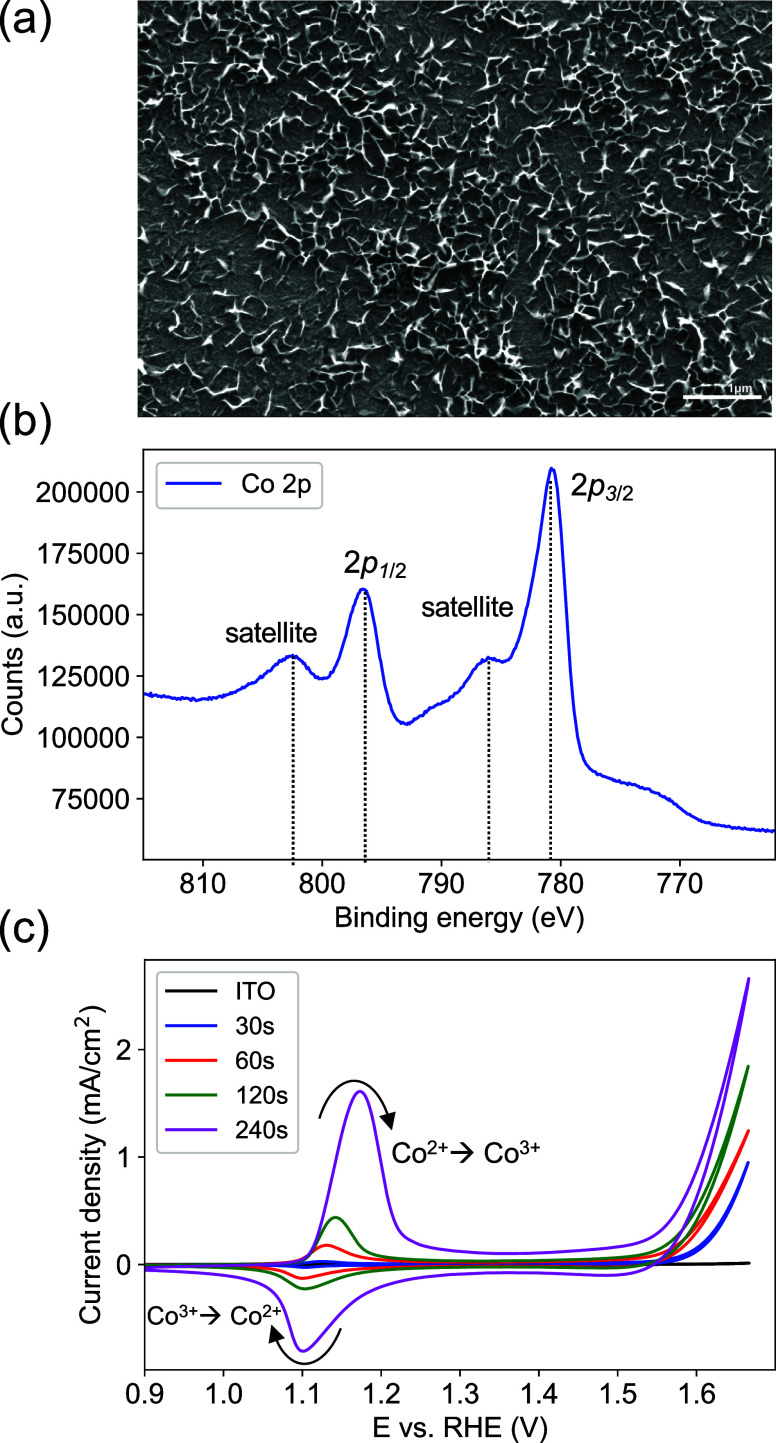
Characterizations of Co(OH)_2_ electrocatalyst film. (a) SEM images of Co(OH)_2_ with 60 s deposition time. The scale bar is 1 μm. (b) High-resolution XPS scan of Co 2p in the as-deposited Co(OH)_2_ sample. (c) Cyclic voltammograms of Co(OH)_2_ films of different deposition times in 0.1 M KOH with a scan rate of 10 mV/s.

A Co(OH)_2_ thin film was prepared via cathodic deposition, and the loading of the catalyst on the conductive substrate was controlled by the deposition time. As shown in Figure 1a, the Co(OH)_2_ film on an ITO substrate exhibits a flaky morphology. The oxidation state of the surface Co metal in the as-deposited film was determined to be Co(II) based on the binding energies of Co 2p_3/2_ in the XPS analysis (Figure 1b), consistent with the oxidation state of Co metal centers in Co(OH)_2_.^29^ This also agrees with the Pourbaix diagram of Co species^30^ in alkaline solutions. Similar to a previous report,^26^ a higher loading of the Co(OH)_2_ layers leads to higher currents of the Co redox wave as well as the OER as demonstrated in the cyclic voltammograms (Figure 1c). More details of the peak analysis of the time-dependent electrodeposited Co(OH)_2_ films are shown in Table S1. Under anodic potentials, Co(OH)_2_ is oxidized to CoOOH as represented by the redox wave of the Co^2+/3+^ couple. As the loading of Co(OH)_2_ increases, this redox wave becomes larger and a higher OER current is observed (Figure 1c). A deposition time of 60 s was chosen to coat the plasmonic Au electrodes with a Co(OH)_2_ layer, ensuring clear redox and OER characteristics while allowing for sufficient light penetration.

**Figure 2:**
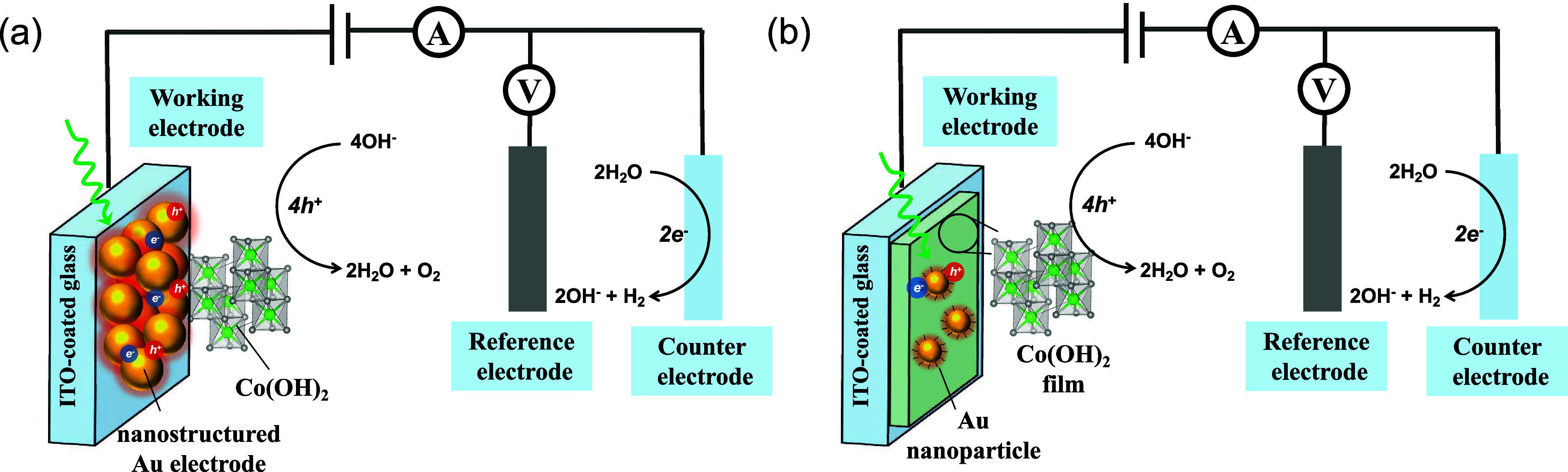
(a) Scheme of Co(OH)_2_ deposited on a plasmonic Au electrode and (b) scheme of free-flowing surfactant-coated Au nanoparticles suspended in an alkaline solution interacting with the Co(OH)_2_ electrode.

The Co(OH)_2_ electrocatalyst was then integrated with plasmonic Au electrodes and Au nanostructures with two types of interfaces. In the first scenario (Figure 2a), a direct interface was created between Co(OH)_2_ and the plasmonic Au nanostructure by electrodepositing Co(OH)_2_ onto electrodeposited plasmonic Au electrodes. The Au electrode serves a dual role as the conductive substrate for the Co(OH)_2_ electrocatalyst and the plasmonic platform. This ensures efficient charge transfer between Au and the ITO contact and a direct interaction between the Co(OH)_2_ electrocatalyst and the Au electrode. As a control system, PEG-coated Au nanoshells were dispersed in the electrolyte, leading to random physical adsorption of Au nanoshells on the surface of the Co(OH)_2_ electrode (Figure 2b). In this case, ITO is the conductive substrate for the Co(OH)_2_ electrocatalyst, and Au nanoshells solely work as the plasmonic enhancer.

**Figure 3:**
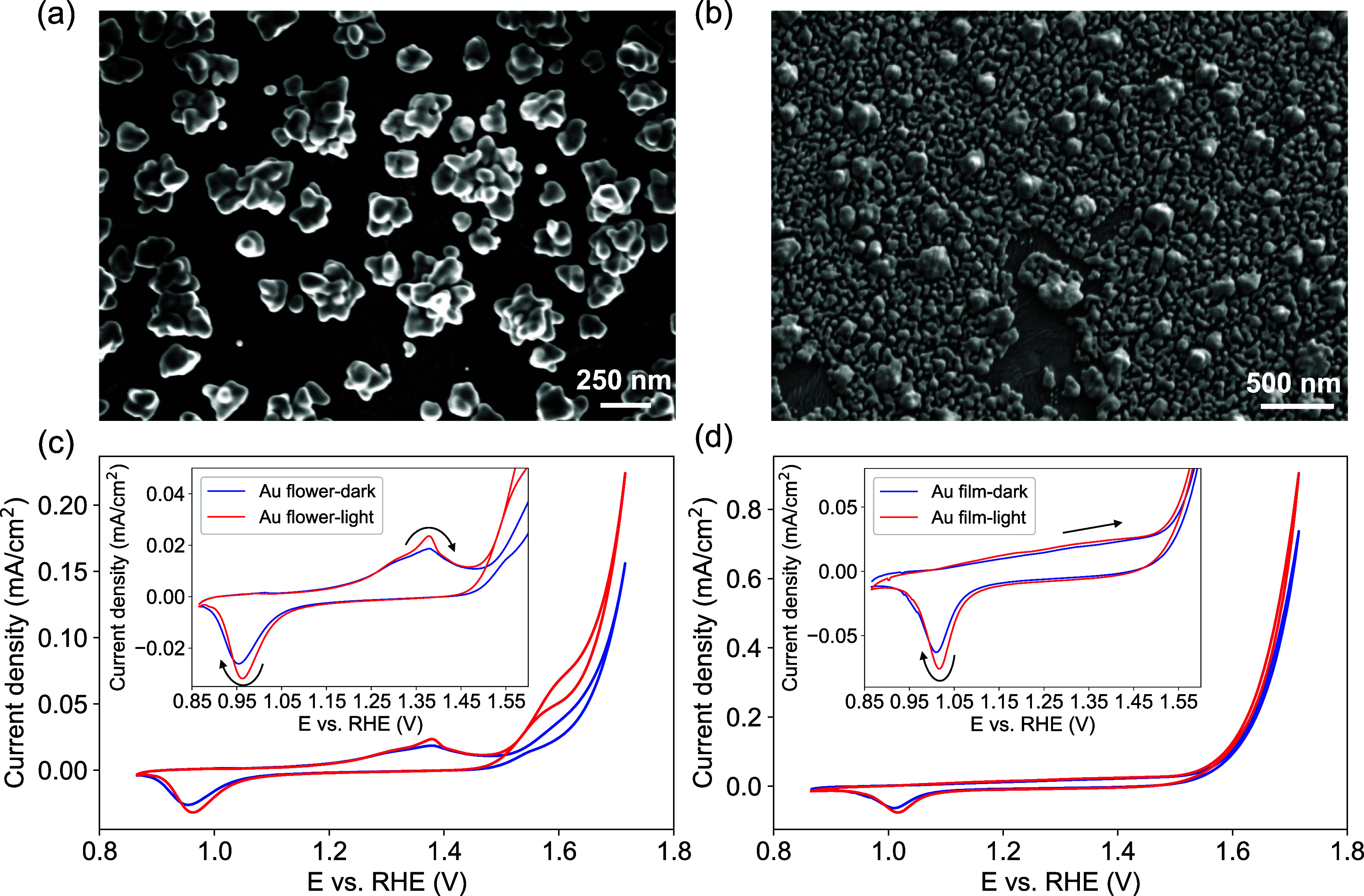
SEM images of (a) Au nanoflower and (b) Au nanofilm electrodes and CVs of (c) a Au nanoflower and (d) a Au nanofilm electrode in the dark and under light illumination. The electrolyte is 0.1 M KOH, and the scan rates are all 10 mV/s. The power intensity of light illumination is 415 mW/cm^2^.

In the first type of interface (Figure 2a), two distinct Au electrode morphologies were prepared via electrodeposition, which are referred to as Au nanoflowers and Au nanofilms (Figure 3a,3b). XPS analysis confirmed that both electrodes consisted of metallic Au (Figure S1). The redox properties of these nanostructured Au electrodes were investigated by using cyclic voltammetry in alkaline electrolytes. The Au nanoflowers exhibited a broad oxidation peak at 1.50 V vs RHE and a reduction peak at 1.05 V vs RHE, whereas the Au nanofilm displayed a less pronounced oxidation peak and a clear reduction peak at 1.12 V vs RHE (Figure 3c,3d). The less cathodic reduction potential observed for the Au nanofilm compared to the Au nanoflowers could be attributed to differences in their electrical conductivity. In addition to the differences in their redox waves, the Au nanofilm also exhibited a smaller Tafel slope for the OER currents (Figure S2). The Tafel analysis was conducted from cyclic voltammetry data with a scan rate of 10 mV/s without applying *iR* compensation. With these two electrodes exhibiting broad absorptions in the visible and near-infrared ranges (Figure S3), their cyclic voltammograms were recorded under continuous light illumination from a solar simulator coupled with a 515 nm long-pass filter. Upon illumination, the reduction peaks of both electrodes shifted anodically and the OER currents increased in the illuminated cyclic voltammograms (Figure 3c,d). The Tafel slopes of the illuminated Au electrodes also decreased (Figure S2), indicating a more efficient OER process. Although Au is not an efficient OER electrocatalyst, illuminating the Au electrodes enhanced the OER efficiency. Similar enhanced water oxidation was observed on an illuminated porous Au electrode and it was proposed that illumination of the Au electrode increases the equilibrium coverage of Au–OH, driven by “hot” charge carriers.^11^

The PEG-coated Au nanoshells with a 660 nm absorption maximum were used in a control experiment. The PEG-coated Au nanoshells were drop cast on an ITO electrode, and the CVs of the physically adsorbed Au nanoshells were measured with and without light illumination. As shown in Figure S4, the intensity of the Au redox waves increased under light illumination, but the peak positions did not shift significantly compared to the plasmonic Au electrodes.

The Co(OH)_2_ layer was subsequently deposited on the surface of the Au electrodes, forming the Au|Co(OH)_2_ interface. As shown in Figure S5, the Au nanofilm|Co(OH)_2_ electrode displayed flaky Co(OH)_2_ sheets decorated with Au nanoparticles, whereas the Au nanoflower|Co(OH)_2_ electrode exhibited only flaky Co(OH)_2_ sheets. Despite the different morphologies between these two electrodes, XPS analysis revealed similar binding energies of Au 4f and Co 2p, confirming the presence of both Au and Co(OH)_2_ in both structures (Figure S5). For the control sample, the Co(OH)_2_ layer was deposited on an ITO substrate and PEG-coated Au nanoshells were introduced into the electrolyte. As the Co(OH)_2_ did not show significant optical absorption in the visible and near-infrared range, the composite electrodes preserved the optical properties of the Au components (Figure S6).

**Figure 4:**
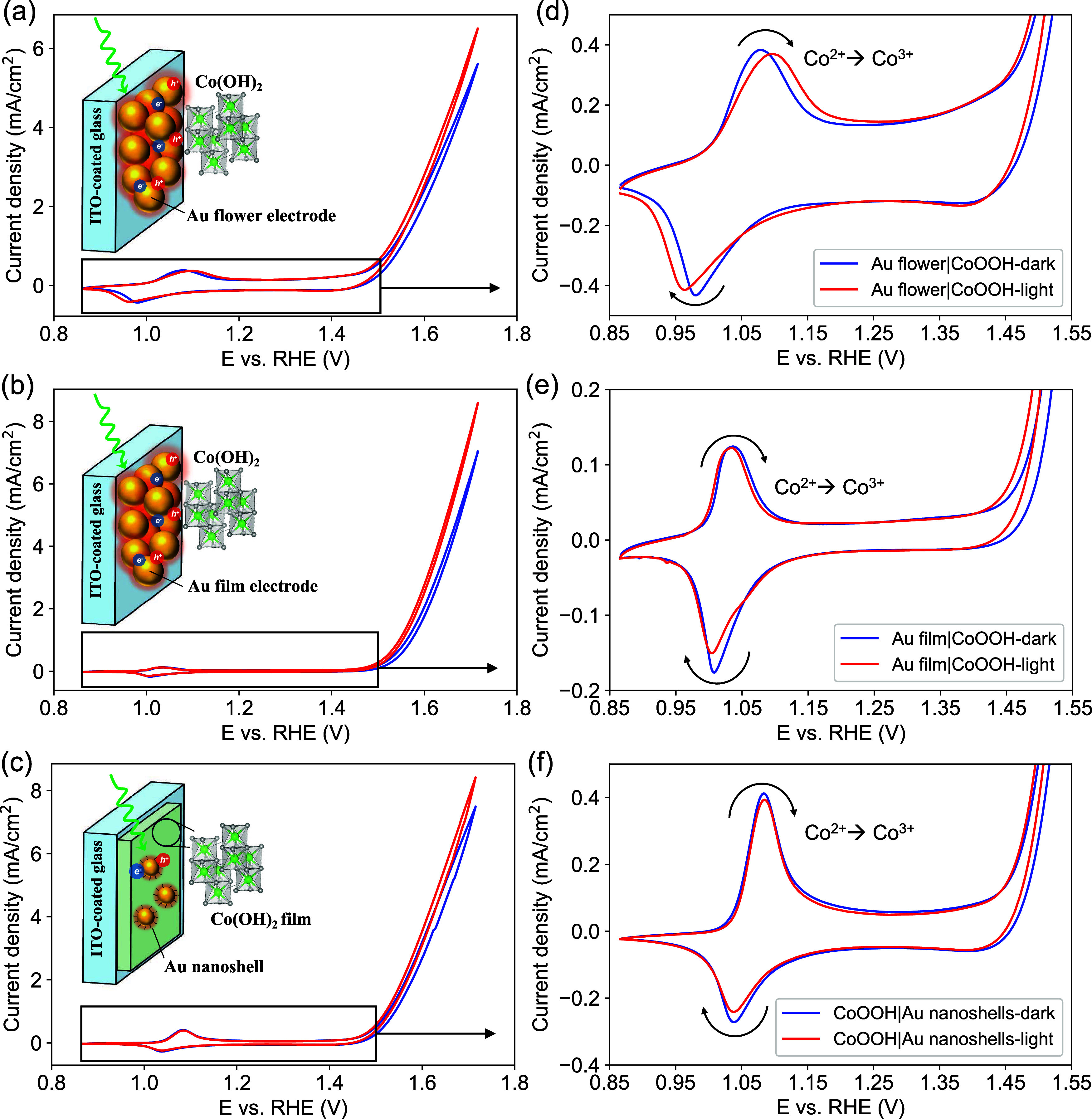
Cyclic voltammograms of Co(OH)_2_ deposited on (a) plasmonic Au nanoflower electrode, (b) Au nanofilm electrode, and (c) ITO substrate and in contact with Au nanoshells in the electrolyte under different illumination conditions. (d–f) Highlight the redox waves of Co species presented in (a–c), respectively. The electrolytes are 0.1 M KOH, and all the scan rates are 10 mV/s.

The Co(OH)_2_ electrode showed the peaks of the Co^2+/3+^ redox couple centered at approximately 1.15 V vs RHE in the Co(OH)_2_/CoOOH transformation (Figure S7). The light illumination does not pose any positive impacts on its redox activities or the performance of the OER performance. As shown in Figure S7, the redox peaks of the Co(OH)_2_ layer slightly diminished under light exposure and this behavior was consistently observed across multiple cycles of alternating dark and light conditions. It is also noted that the Co oxidation peak shifted slightly anodically (Figure S7a). For the Au nanoflower|Co(OH)_2_ and Au nanofilm|Co(OH)_2_ electrodes, the Co redox features are identical to that of the Co(OH)_2_ electrode. The reduction peak of Co cathodically shifted with a decrease in the current upon illumination (Figure 4a,b insets). The larger peak separation of the Co^2+/3+^ redox couple implies that the electron transfer kinetics within the catalyst layer are slightly hindered. For the Co(OH)_2_ electrode interacting with free-flowing Au nanoshells in the solution, the redox waves of Co slightly decreased under illumination but the peak positions did not shift significantly (Figure 4c).

Interestingly, despite the decrease in the redox waves of the Co cations interfaced with illuminated Au nanostructures, the OER currents were enhanced for the Co(OH)_2_ layers in all three electrodes. As shown in Figure 4, the Au nanoflower produced a 26% increase in the OER current at 1.81 V vs RHE, while the Au nanofilm achieved a 14% increase at the same potential with the assistance of light illumination. Further Tafel analysis of the composite electrodes containing plasmonic components in Figure S8 shows that light illumination decreased the Tafel slopes of all of the electrodes. Since light did not improve the OER performance of the Co(OH)_2_ layer (Figure S7), this enhancement was solely attributed to the Au components. Compared with the OER enhancements observed with Au electrodes alone, these enhancement factors were slightly lower, likely due to light blocking caused by the Co(OH)_2_ layer. While the beneficial role of Au as a conductive substrate for the OER has been previously established, and the cooperative interaction of Au and CoOOH has been identified in the electrochemical oxidation of benzyl alcohol,^25,31,32^ plasmonic excitation of the Au components could introduce an additional mechanism to further boost the performance of these systems. In the control experiment where Au nanoshells were added to the electrolyte, the OER current only showed a 6% enhancement at 1.81 V vs RHE (Figure 4c). This implies that plasmonic Au nanostructures as the conductive substrates and a direct interface provide a more significant enhancement via the plasmonic effects.

To better understand the impact of the illumination on the photocurrent response of the electrode systems, the illumination intensity was adjusted. It was found the photocurrents of both electrodes showed a nonlinear dependence on the illumination power intensity (Figure S9). This indicates that the “hot” charge carriers are not the sole factor dominating the enhancement, as “hot” charge carriers are expected to lead to a linear dependence on the power intensity.^33^ To further identify the mechanism by which plasmonic properties enhanced the OER performances of the Co(OH)_2_ electrocatalysts, photocurrent transients of these electrodes were measured under different applied potentials. This approach aims to differentiate between photothermal and photoelectrical (i.e., energetic charge carriers) effects.^17^ Since the photoelectrical effect occurs within a few milliseconds while the heat dissipation due to the photothermal effect takes longer to make an impact, these measurements are expected to help distinguish these two effects.

Chopped illumination experiments were conducted by turning the light on and off every 30 s to measure the current responses of the three electrodes at different applied potentials. As shown in Figures S10–S12, current increases upon light illumination and decreases when the light illumination is turned off at all the applied potentials examined for all three electrodes. The photocurrent of a given cycle is defined as the current difference between the “on” and “off” conditions of illumination at a given applied potential. It can be seen in Figure S10 that the photocurrent transients of Au nanoflower|Co(OH)_2_ exhibited different response rates at different potentials. At 1.56 V vs RHE, which is anodic than the OER onset (Figure S10a), the Au nanoflower|Co(OH)_2_ electrode showed an instantons photocurrent when the light was turned on, followed by a slow increase of current when illuminated. The quick increase of current is attributed to the photoelectrical effect, while the slow component is likely due to the photothermal effect. It agrees with a previous study that plasmon excitation leads to both photothermal and nonthermal enhancements.^34^ The distinct separation of fast and slow responses was less clear at higher anodic potentials (Figure S10). Similar observations were found in the Au nanofilm|Co(OH)_2_ electrode (Figure S11) and Co(OH)_2_|Au nanoshells electrode (Figure S12). It was found that the fast response is more significant for the direct interface comparing two different interfaces of the Au nanoflower|Co(OH)_2_, nanofilm|Co(OH)_2_, and Co(OH)_2_|Au nanoshells electrodes (Figure S13), implying the higher contribution of the photoelectrical effect in the direct interface. In addition, it was estimated based on the current response rates that 33% of the current increases of the Au nanoflower|Co(OH)_2_ come from the photoelectrical effect while the Au nanofilm|Co(OH)_2_ exhibited a lower contribution of 9.6% from the photoelectrical effect at 1.56 V vs RHE (Figure S13). This implies that relative plasmonic contributions from the photothermal and photoelectrical effects could be tuned by engineering the interfaces, electrode architectures, and morphologies.

**Figure 5:**
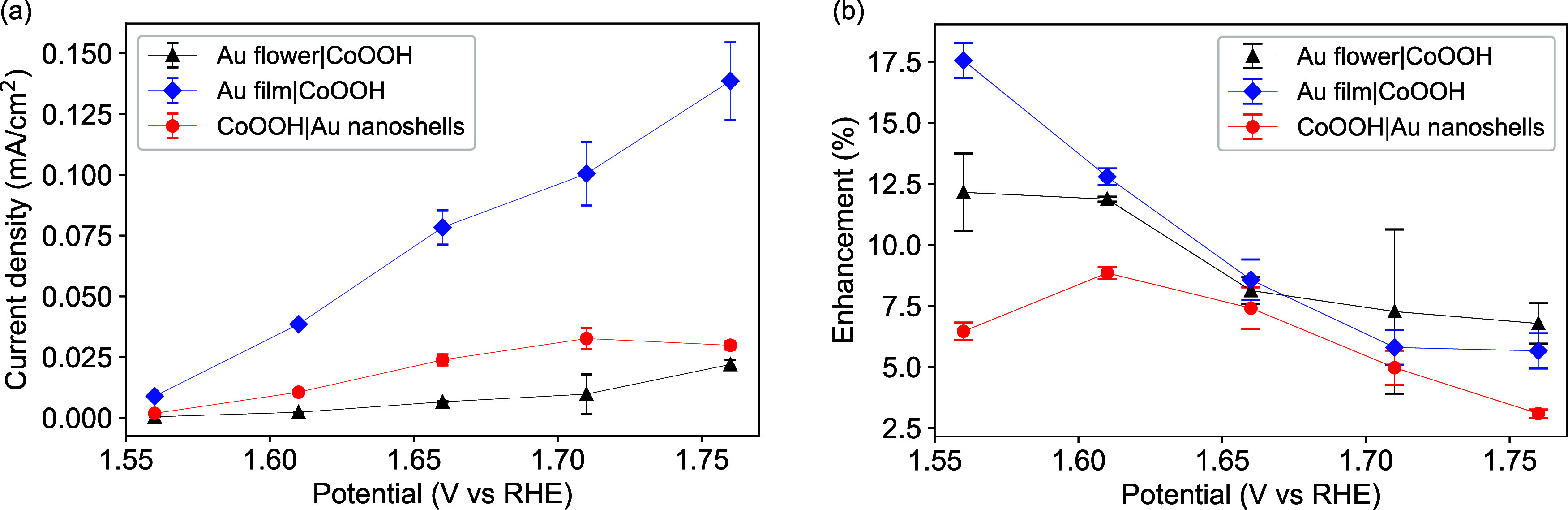
(a) Photocurrent densities and (b) enhancement of currents of a Au nanoflower|Co(OH)_2_ electrode, a Au film|Co(OH)_2_ electrode, and a Co(OH)_2_ electrode with Au nanoshells in the electrolyte of 0.1 M KOH under different applied potentials during chopped illumination. The values are the averages of three different cycles.

Further analysis of the current responses at different potentials under chopped illuminations indicates that when the applied potentials became more anodic, the three electrodes in general exhibited higher photocurrents (Figure 5a). However, the Au nanoshells control electrode first showed an increase and then a slight decrease at 1.76 V vs RHE (Figure 5a). In terms of the current enhancement (photocurrent/current in the dark) at different applied potentials (Figure 5b), it decreased with more anodic potentials for both the Au film and the Au nanoflower electrodes. The Au nanoshell electrode showed a different trend in such enhancements. In short, the plasmonic enhancements are less significant at more anodic potentials (Figure 5b). This might be related to the faster rate of the OER at more anodic potentials, which benefit less from the energetic charge carriers. Comparing different interfaces, the direct interfaces between the plasmonic electrodes and the Co(OH)_2_ electrocatalyst led to higher enhancement factors.

The photothermal effect was further investigated with a bulk electrolyte heating control experiment. As described by the Nernst equation, the change in the electrolyte temperature will change the reaction kinetics and the equilibrium potential of a redox couple. The electrolyte was heated to change the electrolyte temperature in the range of 45–80 °C. As expected, it was observed that increasing the temperature causes the redox peaks of Co and Au to shift, with the oxidation peaks moving to less anodic potentials and the reduction peaks shifting to less cathodic potentials (Figure S14). The redox waves of the illuminated plasmonic Au electrodes followed the same trend (Figure 3c,3d), which implies the effect of photothermal heating. However, the Co redox peaks in the illuminated Au nanoflower|Co(OH)_2_ electrode shifted in the opposite direction if the photothermal effect is the major plasmonic enhancer. We hypothesize that the impact of light illumination of the redox wave shift of the Co(OH)_2_ layer (Figure S7a) shadows the effect of the heating effect.

**Figure 6:**
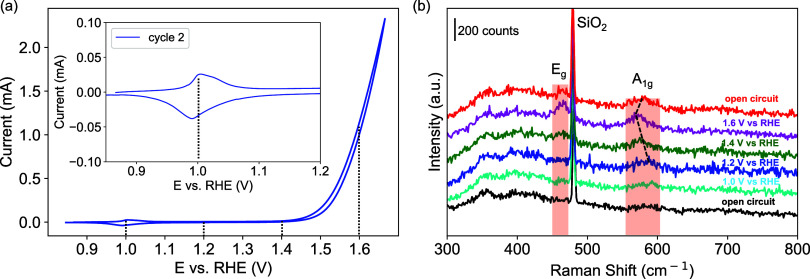
(a) Cyclic voltammogram of Co(OH)_2_ film on a Au film substrate in 0.1 M KOH; (b) EC-Raman spectra of the same Co(OH)_2_ film at different applied potentials in 0.1 M KOH electrolyte. The scan rate in the cyclic voltammogram is 10 mV/s. The objective lens for the Raman measurements is 10×.

To further investigate the dynamics of the Co(OH)_2_ electrocatalyst, we performed *operando* EC-Raman spectroscopy measurements. The electrodeposited Co(OH)_2_ electrocatalyst film on a Au film substrate showed peaks at ∼530 and 590 cm^–1^ in the Raman spectrum with the laser intensity of 0.6 mW (Figure S15a). The peak at ∼530 cm^–1^ is characteristic of Co(OH)_2_, corresponding to the Co–O (A_g_) symmetric stretching mode.^35^ The broad Raman band at 590 cm^–1^ can be attributed to amorphous CoO_*x*_.^36^ When the laser intensity increased from 0.6 W to 6 mW with a reduced acquisition time (Figure S15a), new peaks appeared with a prominent one at ∼680 cm^–1^. This peak is commonly identified as the Co–O (A_1g_) stretching mode in Co_3_O_4_,^35^ and the Raman spectrum of commercial Co_3_O_4_ powder (Figure S15c) confirmed this assignment. This phase transition of Co(OH)_2_ to Co_3_O_4_ is likely induced by laser heating. The substrate effect was also investigated, and it was found that Co(OH)_2_ electrocatalyst films deposited on Au and ITO substrates underwent the same phase transition at the laser intensity of 6 mW (Figure S15b). To avoid laser heating, the *operando* EC-Raman analysis of the Co(OH)_2_ electrocatalyst in 0.1 M KOH electrolyte was conducted with 0.6 mW laser intensity. The EC-Raman peaks of the Co(OH)_2_ electrocatalyst in the electrolyte (Figure 6b) are relatively weaker compared to the dry measurements, as the electrolyte attenuated the signals. The SiO_2_ peak at 480 cm^–1^ comes from the glass substrate^37^ beneath the Au film (Figure 6b). The Raman spectrum of the as-deposited Co(OH)_2_ electrocatalyst in the electrolyte under open circuit conditions was first collected. Following this acquisition, the electrocatalyst was held at different potentials via chronoamperometry (CA) experiments while acquiring the Raman spectra. It could be seen in Figure 6b that when oxidizing potentials were applied, a new peak at ∼460 cm^–1^ was observed from 1.4 V vs RHE and the broad peak at ∼590 cm^–1^ shifted to lower frequencies. These two features could be attributed to the E_g_ and A_g_ vibrational modes of CoO_2_ units in CoOOH.^38^ This supports previous findings that the active phase of the Co(OH)_2_ electrocatalyst is CoOOH at potentials for the OER.^28^ When the CoOOH_2_ electrocatalyst was left at open circuit conditions following the oxidation sequence, the E_g_ peak became smaller, and the A_g_ peak shifted back to a higher frequency. This indicates that maintaining the CoOOH phase requires an applied oxidizing potential. In a control experiment, the Co(OH)_2_ electrocatalyst, which partially transitioned to Co_3_O_4_ due to laser heating, was also investigated, and a similar transition to CoOOH at an oxidizing potential was also observed (Figure S16). We therefore propose that the plasmonic effects from the Au primarily enhance the OER by interacting with the conductive CoOOH layer.

## Conclusions

In summary, we developed three distinct interfaces between plasmonic Au electrodes or nanoparticles and the Co(OH)_2_ OER electrocatalyst. EC-Raman studies confirmed that the active phase of the electrocatalyst during the OER is CoOOH. It was demonstrated that illuminated plasmonic Au electrodes could enhance the performance of the OER either independently or through interaction with the Co(OH)_2_ electrocatalyst. Interestingly, although the redox properties of Co(OH)_2_ were not significantly improved by the plasmonic Au electrodes/nanostructures, the OER performance exhibited a notable enhancement. Photocurrent transients revealed that the plasmon-driven enhancement is due to both photothermal and energetic charge carrier effects, with the photothermal effect playing a more dominant role. However, the contributions from each effect varied depending on the interface between the plasmonic Au components and the electrocatalyst. A direct interface between the plasmonic Au electrode and the electrocatalyst proved more effective than randomly adsorbed free-flowing Au nanoparticles in facilitating transfer of energetic holes. Future work will focus on further correlating the redox activity of the OER electrocatalyst with its OER performance, and the impact of the localized photothermal heating in plasmon-mediated electrocatalytic reactions.

## Supporting Information Available

Table of electrochemical properties of Co(OH)_2_ from different deposition time; XPS spectra of plasmonic Au electrodes; absorbance spectra of all electrodes; SEM images of Au|Co(OH)_2_ electrodes and Au nanoshells; Tafel plots of all electrodes; photocurrent transients of Au|Co(OH)_2_ electrodes at different applied potentials; temperature-dependent cyclic voltammograms of Au|Co(OH)_2_ electrode; Raman spectra of Co(OH)_2_ and Co_3_O_4_ power; EC-Raman spectra of Co(OH)_2_ (PDF)

## References

[ref1] Li A.; Sun Y.; Yao T.; Han H. Earth-Abundant Transition-Metal-Based Electrocatalysts for Water Electrolysis to Produce Renewable Hydrogen. Chem. - Eur. J. 2018, 24 (69), 18334–18355. 10.1002/chem.201803749. 30198114

[ref2] Chatenet M.; Pollet B. G.; Dekel D. R.; Dionigi F.; Deseure J.; Millet P.; Braatz R. D.; Bazant M. Z.; Eikerling M.; Staffell I.; et al. Water Electrolysis: From Textbook Knowledge to the Latest Scientific Strategies and Industrial Developments. Chem. Soc. Rev. 2022, 51 (11), 4583–4762. 10.1039/D0CS01079K. 35575644 PMC9332215

[ref3] Lee H.; Kim J. Y.; Lee S. Y.; Hong J. A.; Kim N.; Baik J.; Hwang Y. J. Comparative Study of Catalytic Activities among Transition Metal-Doped IrO_2_ Nanoparticles. Sci. Rep. 2018, 8 (1), 16777 10.1038/s41598-018-35116-w. 30425306 PMC6233218

[ref4] Li X.; Zhao L.; Yu J.; Liu X.; Zhang X.; Liu H.; Zhou W. Water Splitting: From Electrode to Green Energy System. Nano-Micro Lett. 2020, 12 (1), 131 10.1007/s40820-020-00469-3. PMC7770753 34138146

[ref5] Sundararaman R.; Narang P.; Jermyn A. S.; Goddard W. A. III; Atwater H. A. Theoretical Predictions for Hot-Carrier Generation from Surface Plasmon Decay. Nat. Commun. 2014, 5 (1), 5788 10.1038/ncomms6788. 25511713 PMC4284641

[ref6] Yu S.; Jain P. K. Isotope Effects in Plasmonic Photosynthesis. Angew. Chem., Int. Ed. 2020, 59 (50), 22480–22483. 10.1002/anie.202011805. 32898311

[ref7] Choi C. H.; Chung K.; Nguyen T.-T. H.; Kim D. H. Plasmon-Mediated Electrocatalysis for Sustainable Energy: From Electrochemical Conversion of Different Feedstocks to Fuel Cell Reactions. ACS Energy Lett. 2018, 3 (6), 1415–1433. 10.1021/acsenergylett.8b00461.

[ref8] Zhang C.; Jia F.; Li Z.; Huang X.; Lu G. Plasmon-Generated Hot Holes for Chemical Reactions. Nano Res. 2020, 13 (12), 3183–3197. 10.1007/s12274-020-3031-2.

[ref9] Huang Y.; Liu J.; Cao D.; Liu Z.; Ren K.; Liu K.; Tang A.; Wang Z.; Li L.; Qu S.; Wang Z. Separation of Hot Electrons and Holes in Au/LaFeO_3_ to Boost the Photocatalytic Activities Both for Water Reduction and Oxidation. Int. J. Hydrogen Energy 2019, 44 (26), 13242–13252. 10.1016/j.ijhydene.2019.03.182.

[ref10] Chen D.; Zhang R.; Wang R.; Negro L. D.; Minteer S. D. Gold Nanofiber-Based Electrodes for Plasmon-Enhanced Electrocatalysis. J. Electrochem. Soc. 2016, 163 (14), H1132–H1135. 10.1149/2.0501614jes.

[ref11] Graf M.; Vonbun-Feldbauer G. B.; Koper M. T. M. Direct and Broadband Plasmonic Charge Transfer to Enhance Water Oxidation on a Gold Electrode. ACS Nano 2021, 15 (2), 3188–3200. 10.1021/acsnano.0c09776. 33496564

[ref12] Contreras E.; Nixon R.; Litts C.; Zhang W.; Alcorn F. M.; Jain P. K. Plasmon-Assisted Ammonia Electrosynthesis. J. Am. Chem. Soc. 2022, 144 (24), 10743–10751. 10.1021/jacs.2c01272. 35671395

[ref13] Liu G.; Li P.; Zhao G.; Wang X.; Kong J.; Liu H.; Zhang H.; Chang K.; Meng X.; Kako T.; Ye J. Promoting Active Species Generation by Plasmon-Induced Hot-Electron Excitation for Efficient Electrocatalytic Oxygen Evolution. J. Am. Chem. Soc. 2016, 138 (29), 9128–9136. 10.1021/jacs.6b05190. 27380539

[ref14] Wang C.; Nie X.-G.; Shi Y.; Zhou Y.; Xu J.-J.; Xia X.-H.; Chen H.-Y. Direct Plasmon-Accelerated Electrochemical Reaction on Gold Nanoparticles. ACS Nano 2017, 11 (6), 5897–5905. 10.1021/acsnano.7b01637. 28494145

[ref15] Feng S.; Yang L.; Zhang Z.; Li Q.; Xu D. Au-Decorated CoOOH Nanoplate Hierarchical Hollow Structure for Plasmon-Enhanced Electrocatalytic Water Oxidation. ACS Appl. Energy Mater. 2020, 3 (1), 943–950. 10.1021/acsaem.9b01976.

[ref16] Wilson A. J.; Mohan V.; Jain P. K. Mechanistic Understanding of Plasmon-Enhanced Electrochemistry. J. Phys. Chem. C 2019, 123 (48), 29360–29369. 10.1021/acs.jpcc.9b10473.

[ref17] Zhan C.; Liu B.-W.; Huang Y.-F.; Hu S.; Ren B.; Moskovits M.; Tian Z.-Q. Disentangling Charge Carrier from Photothermal Effects in Plasmonic Metal Nanostructures. Nat. Commun. 2019, 10 (1), 2671 10.1038/s41467-019-10771-3. 31209216 PMC6572789

[ref18] Yu Y.; Sundaresan V.; Willets K. A. Hot Carriers versus Thermal Effects: Resolving the Enhancement Mechanisms for Plasmon-Mediated Photoelectrochemical Reactions. J. Phys. Chem. C 2018, 122 (9), 5040–5048. 10.1021/acs.jpcc.7b12080.

[ref19] Jain P. K. Taking the Heat Off of Plasmonic Chemistry. J. Phys. Chem. C 2019, 123 (40), 24347–24351. 10.1021/acs.jpcc.9b08143.

[ref20] Maley M.; Hill J. W.; Saha P.; Walmsley J. D.; Hill C. M. The Role of Heating in the Electrochemical Response of Plasmonic Nanostructures under Illumination. J. Phys. Chem. C 2019, 123 (19), 12390–12399. 10.1021/acs.jpcc.9b01479.

[ref21] Qiu J.; Wei W. D. Surface Plasmon-Mediated Photothermal Chemistry. J. Phys. Chem. C 2014, 118 (36), 20735–20749. 10.1021/jp5042553.

[ref22] Willets K. A.; Van Duyne R. P. Localized Surface Plasmon Resonance Spectroscopy and Sensing. Annu. Rev. Phys. Chem. 2007, 58 (1), 267–297. 10.1146/annurev.physchem.58.032806.104607. 17067281

[ref23] Zhang Y.; He S.; Guo W.; Hu Y.; Huang J.; Mulcahy J. R.; Wei W. D. Surface-Plasmon-Driven Hot Electron Photochemistry. Chem. Rev. 2018, 118 (6), 2927–2954. 10.1021/acs.chemrev.7b00430. 29190069

[ref24] Zhang Y.; Guo W.; Zhang Y.; Wei W. D. Plasmonic Photoelectrochemistry: In View of Hot Carriers. Adv. Mater. 2021, 33 (46), 2006654 10.1002/adma.202006654. 33977588

[ref25] Li Z.; Yan Y.; Xu S.-M.; Zhou H.; Xu M.; Ma L.; Shao M.; Kong X.; Wang B.; Zheng L.; Duan H. Alcohols Electrooxidation Coupled with H_2_ Production at High Current Densities Promoted by a Cooperative Catalyst. Nat. Commun. 2022, 13 (1), 147 10.1038/s41467-021-27806-3. 35013339 PMC8748678

[ref26] Stevens M. B.; Enman L. J.; Batchellor A. S.; Cosby M. R.; Vise A. E.; Trang C. D. M.; Boettcher S. W. Measurement Techniques for the Study of Thin Film Heterogeneous Water Oxidation Electrocatalysts. Chem. Mater. 2017, 29 (1), 120–140. 10.1021/acs.chemmater.6b02796.

[ref27] Gerken J. B.; McAlpin J. G.; Chen J. Y. C.; Rigsby M. L.; Casey W. H.; Britt R. D.; Stahl S. S. Electrochemical Water Oxidation with Cobalt-Based Electrocatalysts from pH 0–14: The Thermodynamic Basis for Catalyst Structure, Stability, and Activity. J. Am. Chem. Soc. 2011, 133 (36), 14431–14442. 10.1021/ja205647m. 21806043

[ref28] Burke M. S.; Kast M. G.; Trotochaud L.; Smith A. M.; Boettcher S. W. Cobalt–Iron (Oxy)Hydroxide Oxygen Evolution Electrocatalysts: The Role of Structure and Composition on Activity, Stability, and Mechanism. J. Am. Chem. Soc. 2015, 137 (10), 3638–3648. 10.1021/jacs.5b00281. 25700234

[ref29] Cole K. M.; Kirk D. W.; Thorpe S. J. Co(OH)_2_ Powder Characterized by x-Ray Photoelectron Spectroscopy (XPS) and Ultraviolet Photoelectron Spectroscopy (UPS). Surf. Sci. Spectra 2020, 27 (2), 024013 10.1116/6.0000318.

[ref30] Švegl F.; Orel B.; Hutchins M. G.; Kalcher K. Structural and Spectroelectrochemical Investigations of Sol-Gel Derived Electrochromic Spinel Co_3_O_4_ Films. J. Electrochem. Soc. 1996, 143 (5), 1532 10.1149/1.1836675.

[ref31] Strickler A. L.; Escudero-Escribano M.; Jaramillo T. F. Core-Shell Au@Metal-Oxide Nanoparticle Electrocatalysts for Enhanced Oxygen Evolution. Nano Lett. 2017, 17 (10), 040–6046. 10.1021/acs.nanolett.7b02357. 28945433

[ref32] Chakthranont P.; Kibsgaard J.; Gallo A.; Park J.; Mitani M.; Sokaras D.; Kroll T.; Sinclair R.; Mogensen M. B.; Jaramillo T. F. Effects of Gold Substrates on the Intrinsic and Extrinsic Activity of High-Loading Nickel-Based Oxyhydroxide Oxygen Evolution Catalysts. ACS Catal. 2017, 7 (8), 5399–5409. 10.1021/acscatal.7b01070.

[ref33] Linic S.; Christopher P.; Ingram D. B. Plasmonic-Metal Nanostructures for Efficient Conversion of Solar to Chemical Energy. Nat. Mater. 2011, 10 (12), 911–921. 10.1038/nmat3151. 22109608

[ref34] Al-Amin M.; Hemmer J. V.; Joshi P. B.; Fogelman K.; Wilson A. J. Quantification and Description of Photothermal Heating Effects in Plasmon-Assisted Electrochemistry. Commun. Chem. 2024, 7 (1), 70 10.1038/s42004-024-01157-8. 38561493 PMC10984925

[ref35] Yang J.; Liu H.; Martens W. N.; Frost R. L. Synthesis and Characterization of Cobalt Hydroxide, Cobalt Oxyhydroxide, and Cobalt Oxide Nanodiscs. J. Phys. Chem. C 2010, 114 (1), 111–119. 10.1021/jp908548f.

[ref36] Chen Z.; Cai L.; Yang X.; Kronawitter C.; Guo L.; Shen S.; Koel B. E. Reversible Structural Evolution of NiCoO_*x*_H_*y*_ during the Oxygen Evolution Reaction and Identification of the Catalytically Active Phase. ACS Catal. 2018, 8 (2), 1238–1247. 10.1021/acscatal.7b03191.

[ref37] Neuville D. R.; Charpentier T.; Du J. C.; Yue Y. Z.; Blanc W.; Cicconi M. R.; Lancry M.; Ren M. Structure Characterizations and Molecular Dynamics Simulations of Melt, Glass, and Glass Fibers. In Fiberglass Science and Technology; Li H., Ed.; Springer International Publishing:: Cham, 2021; pp 89–216.

[ref38] Moysiadou A.; Lee S.; Hsu C.-S.; Chen H. M.; Hu X. Mechanism of Oxygen Evolution Catalyzed by Cobalt Oxyhydroxide: Cobalt Superoxide Species as a Key Intermediate and Dioxygen Release as a Rate-Determining Step. J. Am. Chem. Soc. 2020, 142 (27), 11901–11914. 10.1021/jacs.0c04867. 32539368

