# Supplementary material for: Interfacial Properties of Gold and Cobalt Oxyhydroxide in Plasmon-Mediated Oxygen Evolution Reaction
Source: J Phys Chem C Nanomater Interfaces. 2025 Jan 6;129(2):1129–37. doi: 10.1021/acs.jpcc.4c06632 (PMC11744795; doi:10.1021/acs.jpcc.4c06632)
Supplement: Supplementary file 1 — jp4c06632_si_001.pdf [file jp4c06632_si_001.pdf]

Supporting Information

Interfacial Properties of Gold and Cobalt Oxyhydroxide in Plasmon-mediated Oxygen Evolution Reaction

Janet Zhen[1], Timothy Lin[1], Tucker Forbes[1], Mark Engelhard[2], Jingjing Qiu[1]*

[1]Department of Chemistry and Biochemistry, San Francisco State University, 1600 Holloway Ave., San Francisco, CA 94132, United States
[2]Energy & Environment, Pacific Northwest National Laboratory, Richland, Washington 99354, United States

*Corresponding author. E-mail: qiu@sfsu.edu

Table S1. Electrochemical properties of $Co(OH)_2$ from different deposition time

Electrode	E_c (V vs RHE)	E_a (V vs. RHE)	j (mA/cm^2) at 1.70 V vs RHE
30 sec deposition	1.13	1.15	0.57
60 sec deposition	1.13	1.16	0.67
120 sec deposition	1.13	1.17	1.30
240 sec deposition	1.13	1.20	1.77

In Table S1, the anodic peak maximum, cathodic peak maximum and the OER current density at 1.70 V vs. RHE were summarized for the $Co(OH)_2$ layer on ITO of different deposition time. As the deposition time increases, the anodic peak shifts anodically. This makes the peak separation between E_c and E_a larger, implying a larger energy barrier for charge and mass transfer of the electrodeposited film. Meanwhile, the OER current increases with the longer deposition time, indicating a higher percentage of electrochemically active metal centers participating in the OER process.

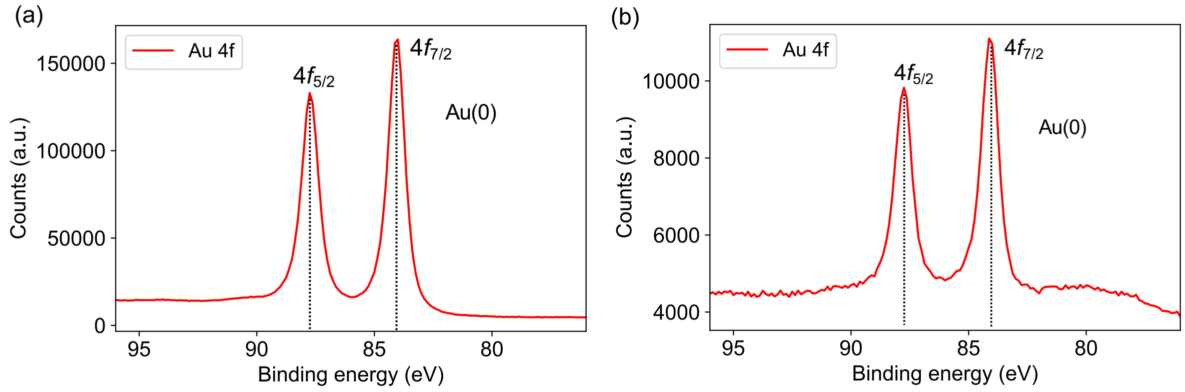

Figure S1. High resolution XPS scans of Au 4*f* of (a) a Au nanofilm electrode and (b) a Au nanoflower electrode.

The binding energies of the Au $4f_{7/2}$ is 84.0 eV for both the Au nanofilm and Au nanoflower electrodes, confirming the oxidation state of Au in both samples to be Au(0).

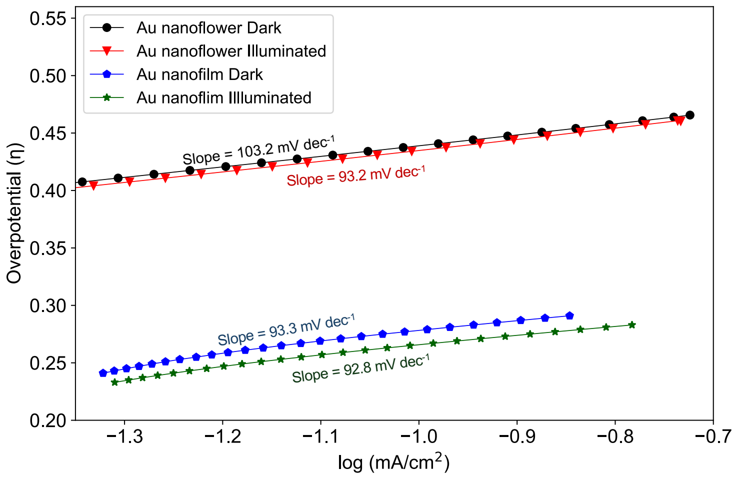

Figure S2. Tafel plots of a Au nanofilm electrode and a Au nanoflower electrode in the dark and under light illumination.

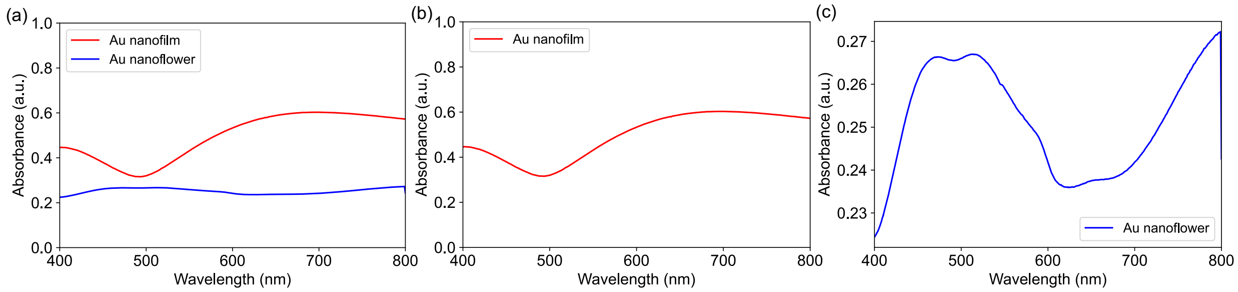

Figure S3. Absorbance spectra of (a) a Au nanofilm electrode and a Au nanoflower electrode on the same scale, (b) a Au nanofilm electrode and (c) a Au nanoflower electrode.

The absorbance spectra of a Au nanofilm electrode and a Au nanoflower electrode were measured with a blank ITO substrate as the baseline. The absorbance spectra were calculated from the transmittance measurements.

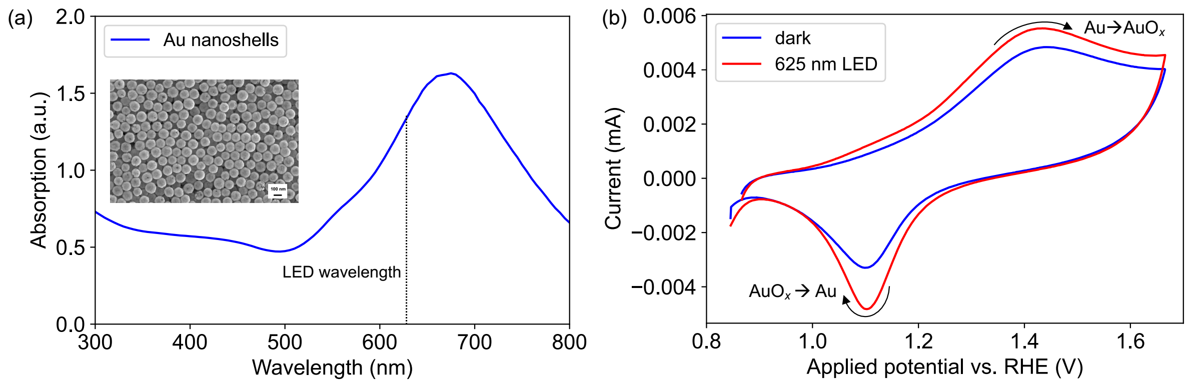

Figure S4. (a) Absorbance of PEG-coated Au nanoshell solution and (b) cyclic voltammograms of PEG-coated Au nanoshells on ITO substrate. The inset in (a) is the SEM image of the Au nanoshells. The electrolyte is 0.1 M KOH and the scan rates are 10 mV/s.

The Au nanoshell solution was drop cast onto an ITO substrate and dried at room temperature. The ITO electrode with physically-adsorbed Au nanoshells was used as the working electrode. It was shown that the illumination from a 625 nm LED light increases the redox activities of the Au but the positions of the redox waves do not shift significantly.

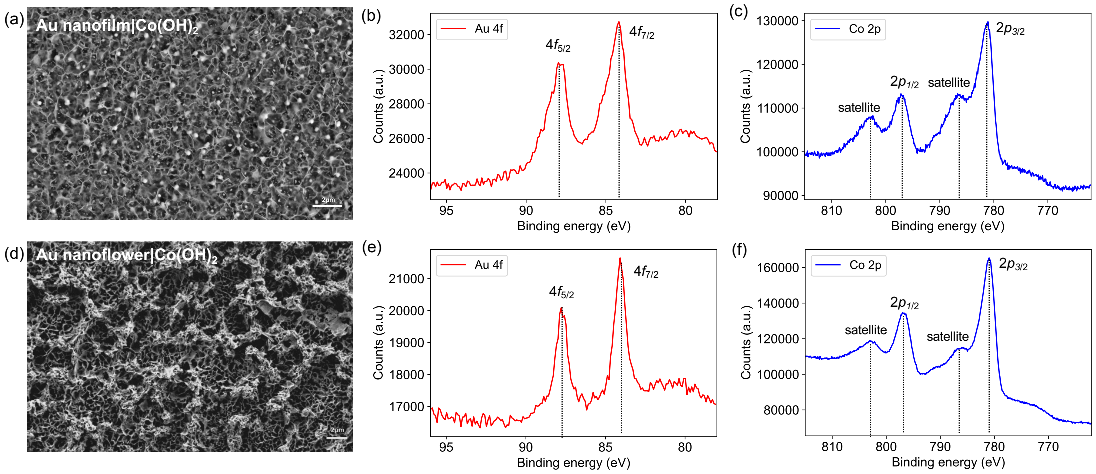

Figure S5. SEM images and XPS analysis of Au 4*f* and Co 2*p* of a Au nanofilm electrode coated with $Co(OH)_2$ (a,b,c) and a Au nanoflower electrode coated with $Co(OH)_2$ (d,e,f).

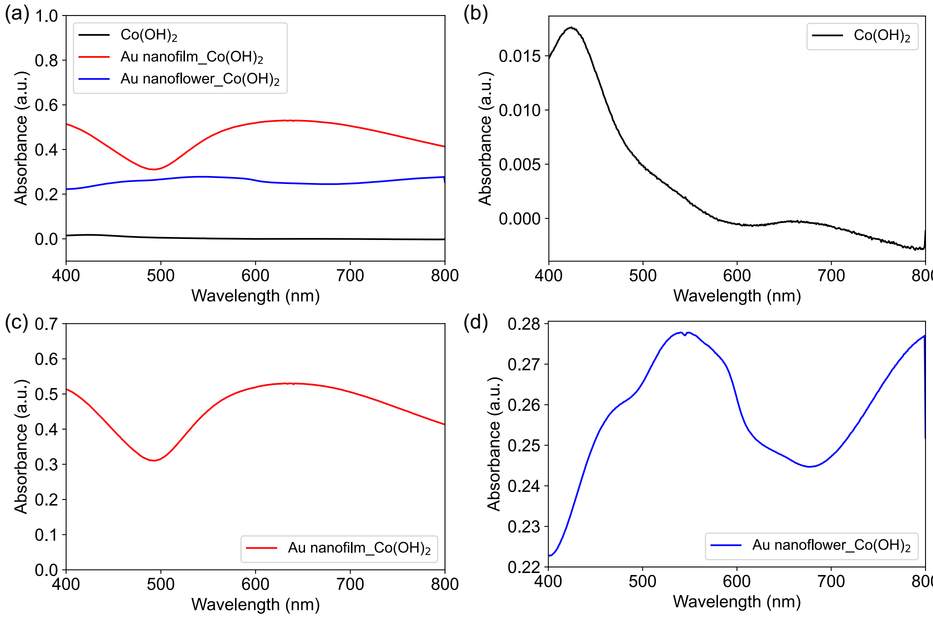

Figure S6. Absorbance spectra of (a) $Co(OH)_2$ electrode, Au nanofilm|$Co(OH)_2$ electrode and Au nanoflower|$Co(OH)_2$ electrode on the same scale and (b) $Co(OH)_2$ electrode, (c) Au nanofilm|$Co(OH)_2$ electrode and (d) Au nanoflower|$Co(OH)_2$ electrode.

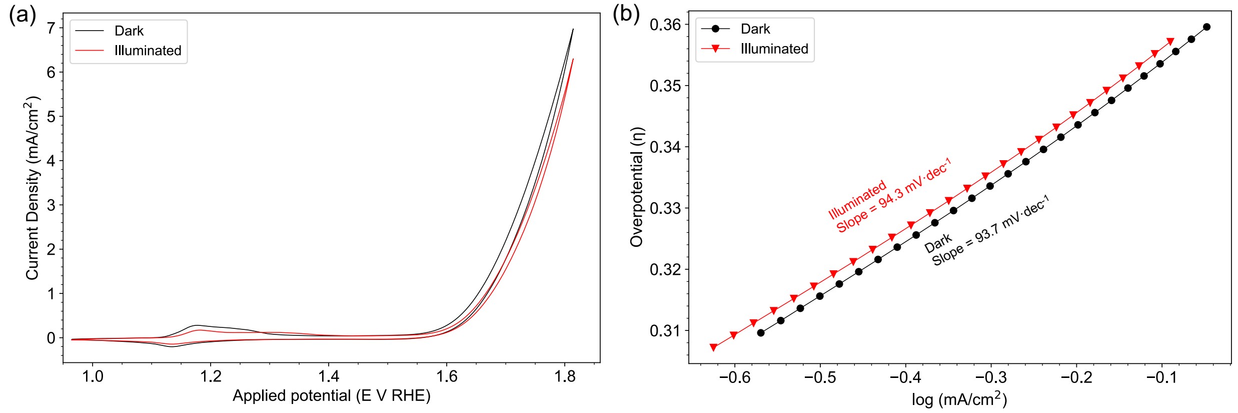

Figure S7. (a) Cyclic voltammograms of $Co(OH)_2$ on ITO substrate in the dark and under light illumination; (b) Tafel plots of $Co(OH)_2$ on ITO substrate in the dark and under light illumination. The electrolyte is 0.1 M KOH and the scan rates are 10 mV/s. The standard deviation of the slopes are 5.1 mV/dec and 2.2 mV/dec for the dark and light measurements, respectively.

The $Co(OH)_2$ electrode was illuminated from the top with a solar simulator coupled with a 515 nm longpass filter, and no enhancement was observed in the illumination experiments. These illumination experiments were repeated for three cycles and enough cooling time was allowed before each cycle. Figure S7 shows the decrease in both cathodic and anodic density of the film under light illumination. The Tafel analysis was also performed, and the illumination condition does not significantly change the Tafel slopes.

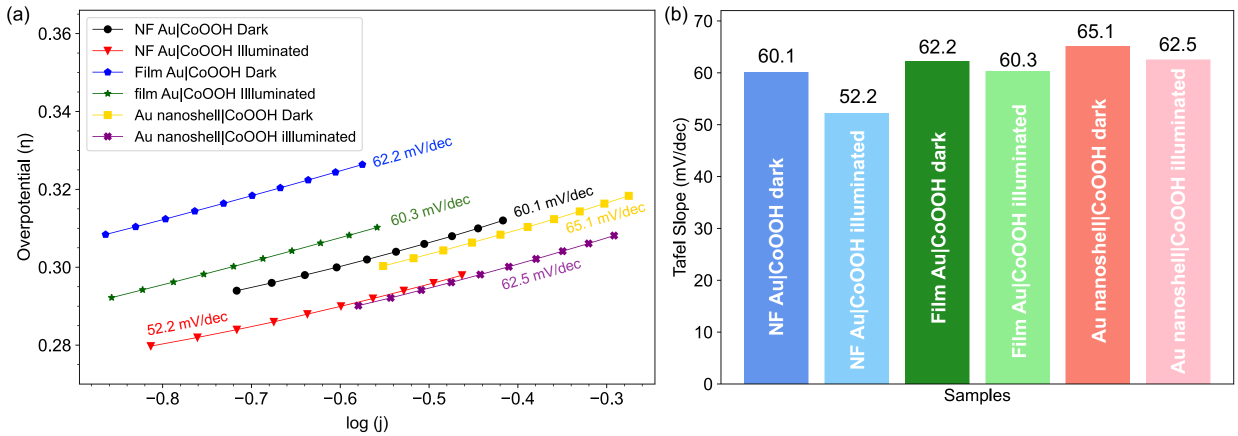

Figure S8. (a) Tafel plots of different Au|CoOOH electrodes in the dark and under light illumination and (b) Bar plots of the Tafel slopes of different samples. The standard deviations of the Tafel slopes are in the range of 3.5-6.5 mV/dec.

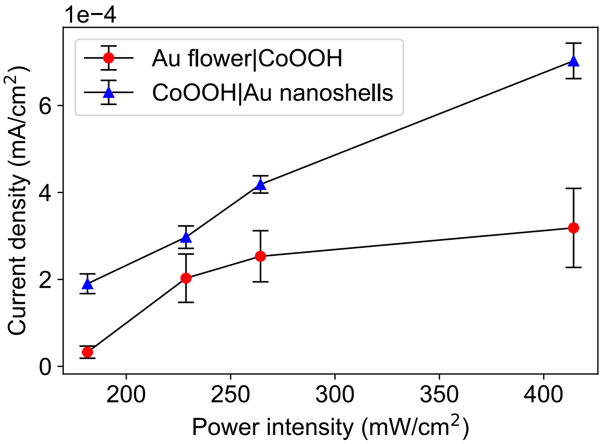

Figure S9. Power-dependent photocurrents of Au nanoflower|CoOOH and CoOOH|Au nanoshells electrodes at 1.56 V vs RHE.

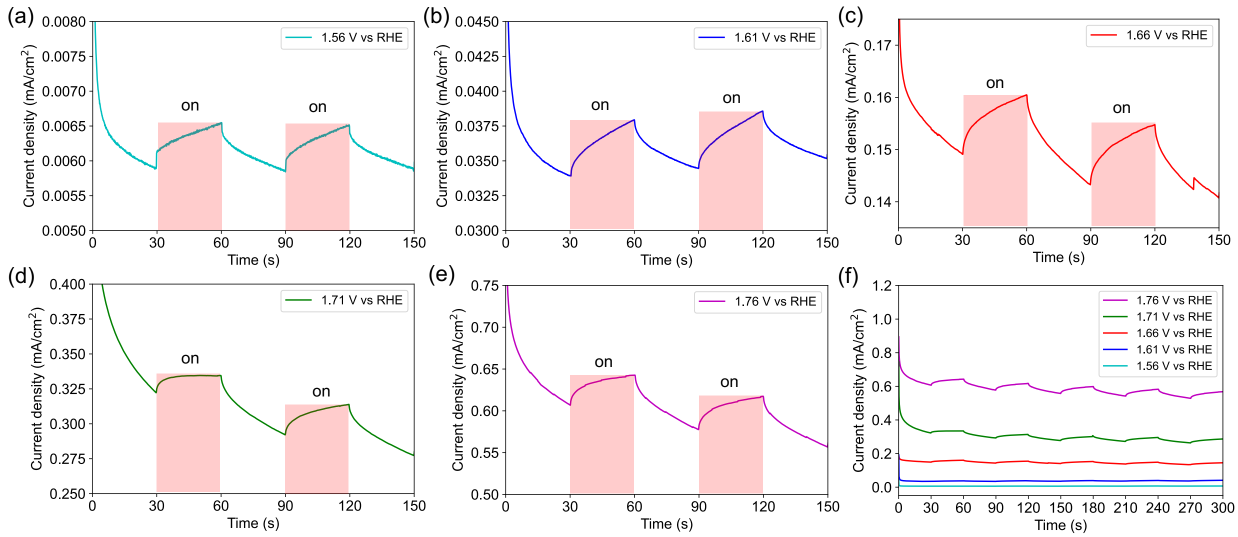

Figure S10. Photocurrent transients of Au nanoflower|$Co(OH)_2$ electrode, and its photocurrents at applied potentials of (a) 1.56 V vs. RHE, (b) 1.61 V vs. RHE, (c) 1.66 V vs. RHE, (d) 1.71 V vs. RHE, (e) 1.76 V vs. RHE and (f) overall of all photocurrent responses under chopped light in 0.1 M KOH.

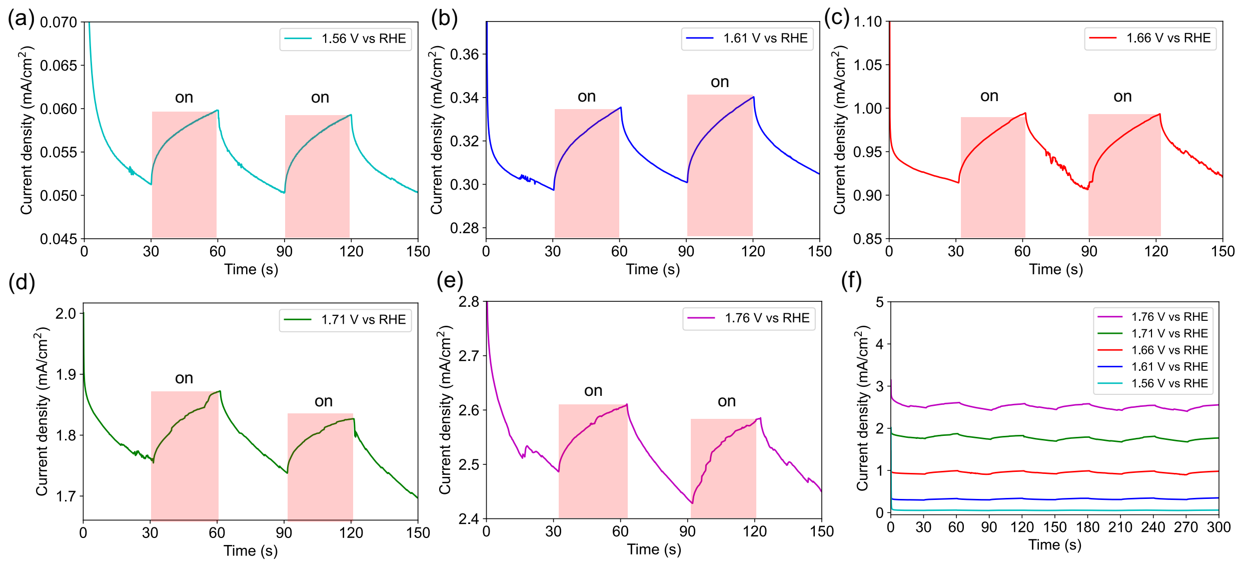

Figure S11. Photocurrent transients of Au nanofilm|$Co(OH)_2$ electrode, and its photocurrents at applied potentials of (a) 1.56 V vs. RHE, (b) 1.61 V vs. RHE, (c) 1.66 V vs. RHE, (d) 1.71 V vs. RHE, (e) 1.76 V vs. RHE and (f) overall of all photocurrent responses under chopped light in 0.1 M KOH.

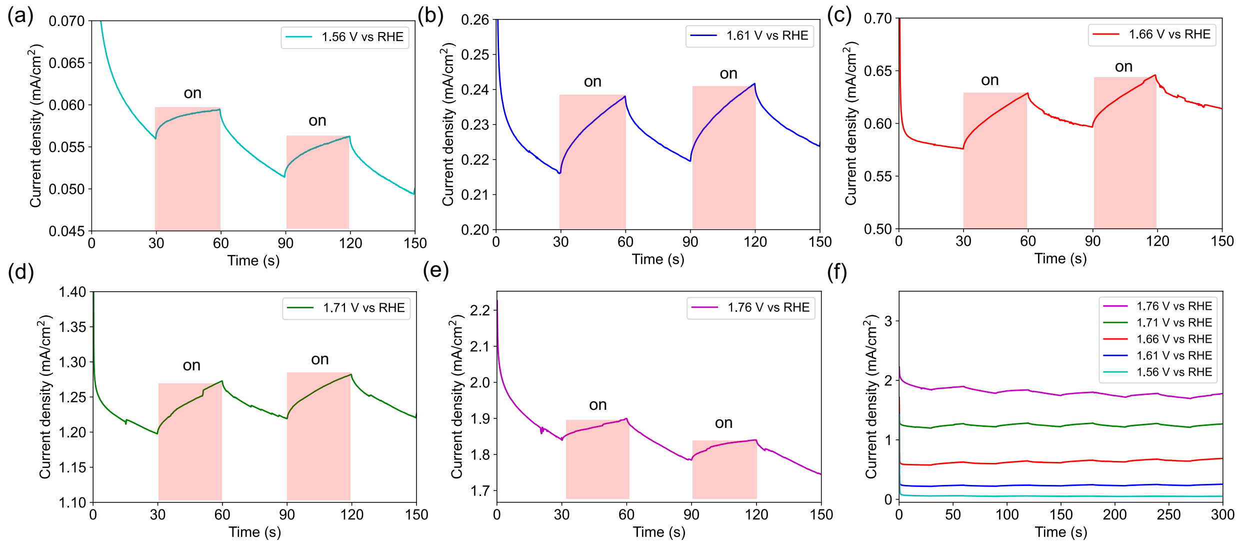

Figure S12. Photocurrent transients of $Co(OH)_2$|Au nanoshells electrode, and its photocurrents at applied potentials of (a) 1.56 V vs. RHE, (b) 1.61 V vs. RHE, (c) 1.66 V vs. RHE, (d) 1.71 V vs. RHE, (e) 1.76 V vs. RHE and (f) overall of all photocurrent responses under chopped light in 0.1 M KOH.

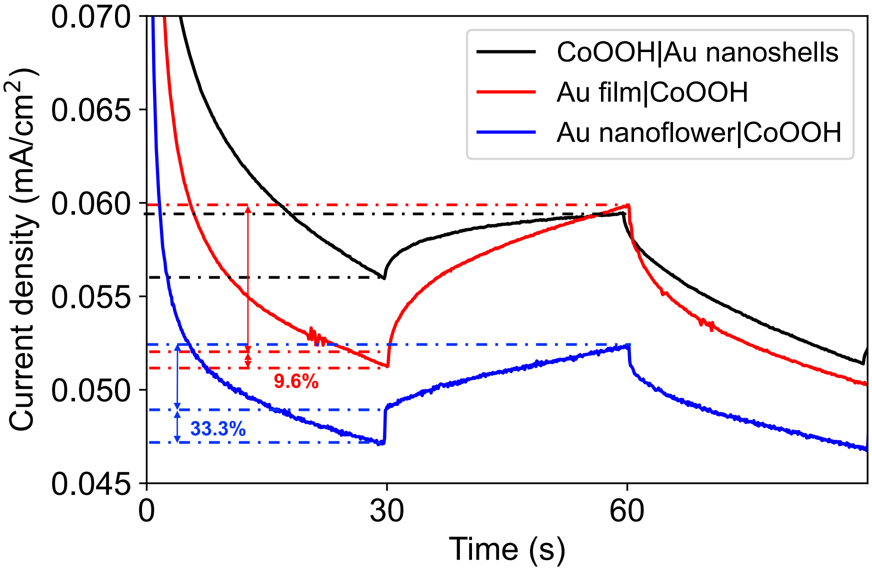

Figure S13. Photocurrent transients of three electrodes at 1.56 V vs. RHE in 0.1 M KOH under chopped illumination. Light is turned on at 30 second and turned off at 60 second. The contributions of the fast responses are marked in the figure.

The first derivative of the photocurrent transient was analyzed within the time interval of 28 to 35 seconds, which represents the rate of current change. In this region, the first derivative initially increases, reaches a maximum value, and then decreases. This local maximum was identified as the turning point between the fast and slow response phases.

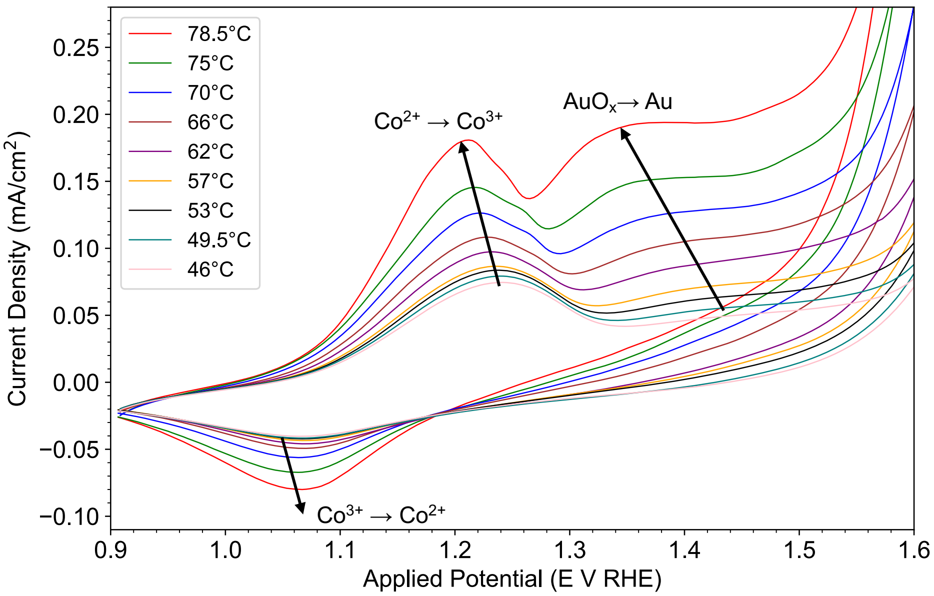

Figure S14. Cyclic voltammograms of $Co(OH)_2$ with Au nanoshells in 0.1 M KOH at different electrolyte temperatures. The scan rates are 10 mV/s.

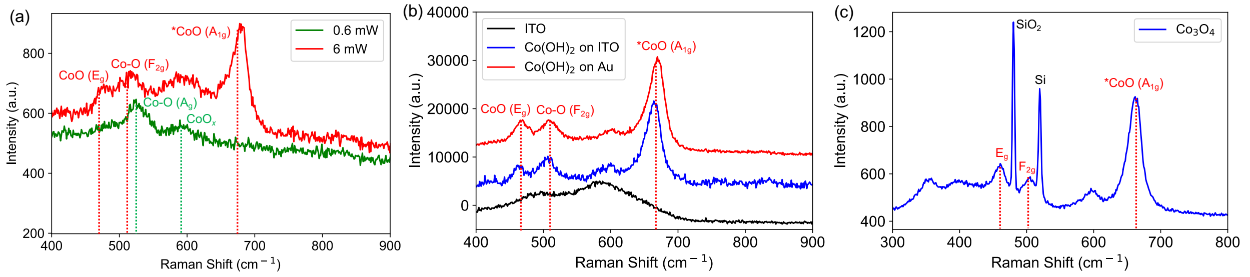

Figure S15. Raman spectra of (a) the dry as-deposited $Co(OH)_2$ electrocatalyst film (60 second deposition time) on a Au film substrate with the 532 nm laser intensity of 0.6 mW (acquisition time 30 s) and 6 mW (acquisition time 3 s); (b) the dry $Co(OH)_2$ electrocatalyst film on Au and ITO substrates with the 532 nm laser intensity of 6 mW (acquisition time 30 s); (c) commercial Co_3O_4 power on Si wafer with the 532 nm laser intensity of 6 mW. The grating used is 1800/mm and the objective lens is 100x.

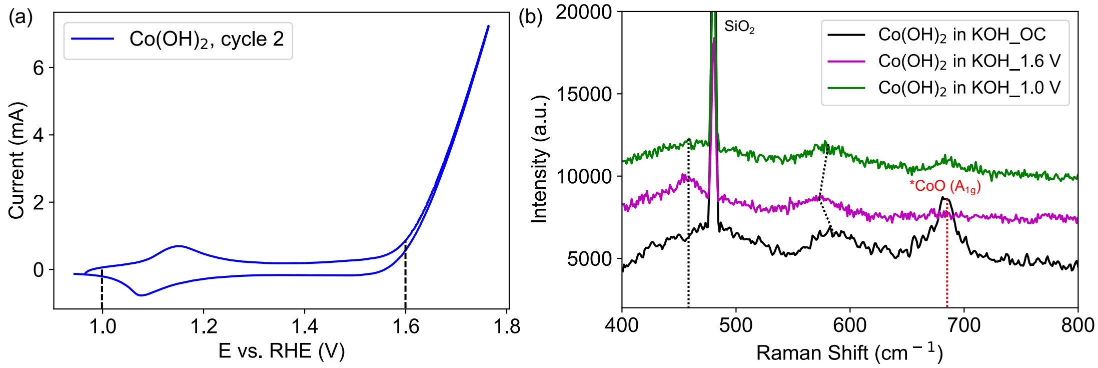

Figure S16. (a) Cyclic voltammogram of $Co(OH)_2$ film on an ITO film substrate in 0.1 M KOH; (b) EC Raman spectra of the same $Co(OH)_2$ film at two applied potentials in 0.1 M KOH electrolyte. The scan rate in the cyclic voltammogram is 20 mV/s. The grating used is 1800/mm and the objective lens for the EC Raman measurements is 10x.
